# The Relationship Between State Loneliness and Depression Among Youths During COVID-19 Lockdown: Coping Style as Mediator

**DOI:** 10.3389/fpsyg.2021.701514

**Published:** 2021-09-14

**Authors:** Yayun Zhang, Lin Huang, Yuejia Luo, Hui Ai

**Affiliations:** ^1^Shenzhen Key Laboratory of Affective and Social Neuroscience, Magnetic Resonance Imaging Center, Center for Brain Disorders and Cognitive Sciences, Shenzhen University, Shenzhen, China; ^2^Center for Emotion and Brain, Shenzhen Institute of Neuroscience, Shenzhen, China

**Keywords:** COVID-19, loneliness, depression, coping style, mediator

## Abstract

The coronavirus disease (COVID-19) pandemic has had a great impact on public mental health. However, loneliness during the lockdown related to depression and whether the relationship would be mediated by coping style or whether sex moderates loneliness and coping style are not clear. The study aimed to examine the mediating role of coping style in the relationship between state loneliness and depression as well as the moderating role of sex in the relationship between state loneliness and coping styles during the COVID-19 lockdown. Participants were 337 college students in China during the COVID-19 pandemic (January–February 2020). States of depression and anxiety, state loneliness, and coping styles with COVID-19 were measured. The results show that loneliness was an effective predictor of depression during self-quarantine. Moreover, coping style mediated the relationship between state loneliness and depression although sex did not moderate the relationship between state loneliness and coping style. Youths were inclined to use more positive coping strategies than negative coping strategies. Our results indicate less loneliness is an effective way to relieve depression, and coping strategies, especially the positive ones, are important for youths to prevent depression and loneliness during the lockdown of the COVID-19 pandemic.

## Introduction

A severe pandemic of infectious diseases suddenly broke out in Wuhan, Hubei Province, China, in December 2019, and it was named the novel coronavirus disease (COVID-19) by the World Health Organization later. The pandemic had a huge impact on people’s mental health and caused problems, such as panic, anxiety, and depression. Moreover, an extended lockdown policy due to COVID-19 has had a huge effect on college students. College education not only puts emphasis on the training of students’ academic skill, but also emphasizes the cultivation of students’ practical ability. However, the extended lockdown has limited their social practice as well as social activities. In terms of age, they are adults, but not mature. In terms of social development, college students are sensitive, impulsive, dependent, and not good at dealing with frustrations. They are more likely to experience emotional problems than people at other developmental stages, which is not profitable for them to go through the pandemic and adjust their lifestyles.

### Depression

Depression is characterized by continuous low mood and anhedonia (De Fruyt et al., 2020). Researchers have focused on depression among college students for a long time and found that more than half of college students report moderate or severe depression (Cramer and Neyedley, 1998; Killeen, 1998; Alorani and Alradaydeh, 2017). Besides this, stress is a situation in which the specific social environment threatens the physical and mental health of individuals (Grant et al., 2003). Such a situation may be a short-term environmental event or a long-term life event. The outbreak of a major pandemic is a typical public health emergency, which seriously threatens the safety of people. People often respond to stress usually with generation or aggravation of depression. Since 2000, China has experienced two major pandemic disasters: the SARS pandemic in 2003 and the H1N1 influenza virus in 2009. Tone (2004) found that depression under acute stress was different from normal depression with more harm. During SARS, 25.38% of medical college students were depressed (Liu et al., 2004). Ding et al. (2011) report that 34.88% of unquarantined college students suffered depression during the influenza (H1N1) outbreak. The depression of college students in the stressful social environment conforms to the general psychological characteristics of the postdisaster population. Hence, exploring the mechanism of depression and its interaction with other mental health variables is helpful to provide a scientific basis for psychological counselors to provide counseling services to college students during the COVID-19 pandemic.

What’s more, anxiety is an innate, survival-oriented stress response of human beings in the face of environmental stress, and it usually occurs when an individual is faced with threats (Beck and Stanley, 1997). Anxiety and depression are common adverse emotional reactions in a stressful social environment, and they often coexist. Therefore, to explore the relationship between coping style, loneliness, and depression effectively, anxiety is added as a covariate.

### Loneliness

Loneliness is a common negative emotion. Fitts et al. (2009) define it as an emotion when an individual experiences a discrepancy between expectation and what they currently perceive. When individuals are not satisfied with their interpersonal relationships, they feel lonely with the perceived gap between what they expected and the objective level in life. It is shown that (1) loneliness stems from dissatisfaction with relationships; (2) loneliness is a subjective feeling, and when someone is isolated, they do not necessarily feel lonely; and (3) loneliness is a negative emotional experience (Baumeister and Leary, 1995; Killeen, 1998).

According to the duration of loneliness, researchers divide it into two types: state and trait loneliness. The former is short term and caused by specific factors, and the latter is long term and related to personality factors. State loneliness can change as the environment changes. For example, students may experience loneliness when they transfer to another school. Trait loneliness is chronic and caused by a prolonged perception of poor relationships. State loneliness is closely related to trait loneliness. Reconnection motivation indicates that, when individuals are disgusted with state loneliness, they are prompted to reconnect with others (Qualter et al., 2015). In short, loneliness affects the connection between individuals and people around them. Tung et al. (2019) report the severity of loneliness increases significantly after exposure to violent neighborhoods with high crime rates and being forced to stay at home for safety. Maharani et al. (2019) find that hearing impairment is positively correlated with loneliness with a longitudinal study. Adamczyk (2016) finds that support from family and significant others can alleviate loneliness among college students. These indicate that, when people’s social behaviors are restricted and their social experiences are poor or when they encounter unpleasant events, they feel lonely. Therefore, it is particularly important to explore the loneliness of college students and its interaction with negative emotions, such as depression states caused by the restriction policy during the pandemic.

### Coping Style

Coping style refers to a mode in which individuals adjust their cognition and behavior patterns to alleviate negative feelings when they are under stress (Compas and Boyer, 2001). It is divided into positive and negative coping (Lazarus and Folkman, 1984). When under stress, individuals using positive coping styles may deal with their negative emotions by adopting positive cognition and seeking help (Lazarus and Folkman, 1984). Positive coping styles include strategies such as “Talk to people and pour out your inner troubles” and “Ask for advice from friends, relatives or classmates,” and these make individuals focus on solving problems and relieving their negative emotions, such as anxiety and depression. The negative coping style is defined as the negative adaptive adjustment by individuals when they realize that their interaction with the surrounding environment may bring some load to them or even exceed the resources they own (Lazarus, 2000). Negative coping styles include strategies such as “Try to take a break or vacation from your troubles for a while” and “Relieve by smoking, drinking, taking medicine, and eating,” and these may not solve the problem properly, and these unresolved difficulties may further bring emotional distress to individuals.

### The Present Study

Researchers have tried to explain the relationship between loneliness and depression from a theoretical perspective as well as an empirical perspective. The deficiency hypothesis of loneliness proposes that individuals with strong loneliness are not good at interpersonal interaction, leading to unsatisfied emotional needs and negative emotions, such as depression and anxiety (Yao et al., 2014). Moreover, social support theory proposes that intense loneliness is caused by the lack of social support, and long-term, high-intensity loneliness could further induce depression (Huang et al., 2019). Except for theoretical indication, previous empirical studies also demonstrate the association between depression and loneliness. A recent meta-analysis summarizes 14 empirical studies and supports the intense positive relationship between loneliness and depression and reports that it is difficult for individuals with high loneliness to relieve depression. Students who live alone or have a bad relationship with people around report severe depression (Shao et al., 2020). Furthermore, studies show that loneliness stably predicts depression and can be an antecedent risk factor for depression (Qualter et al., 2010; Vanhalst et al., 2012; Matthews et al., 2016; Kraav et al., 2021).

The relationship between loneliness and coping style has also been explored before. One study found that teenagers with more loneliness were more likely to use negative coping styles (Van Buskirk and Duke, 1991). Apart from a positive correlation between negative coping and loneliness, a negative correlation between positive coping and loneliness has also been found (Zhao et al., 2017). Coping style also mediates the association between loneliness and other variables, such as self-esteem (Zhao et al., 2017) and adjustment (Quan et al., 2014). In short, a strong association between loneliness and coping style has been found. However, less is known about how the coping style is related to state loneliness caused by lockdown during the COVID-19 pandemic. In addition, researchers have found a strong link between coping style and depression. In a longitudinal study, adolescents who use a negative coping style were found to have more depressive symptoms after 2 years (Seiffge-Krenke and Klessinger, 2000). A negative coping style is positively associated with depression (Boerboom et al., 2014; Abdollahi et al., 2018) and is a main predictor of depression (Mahmoud et al., 2012). Individuals who use positive coping report less depression (Donatti et al., 2017). Hence, it is interesting to explore the relationship between coping style and depression under lockdown during the pandemic.

Furthermore, studies investigate the relationship between coping style, state loneliness, and depression. It is reported that loneliness could hinder the use of positive coping strategies, resulting in a higher probability of depression among Spanish college students (Liang et al., 2019). Only one study split coping styles into positive and negative strategies in teenagers and found coping styles, such as rumination and problem-solving, could both mediate the relationship between loneliness and depression (Zhang et al., 2019). However, whether positive and negative coping styles play a role in the relationship between state loneliness caused by social quarantine and constant high-level depression due to uncertainty during the pandemic in youths is rarely studied.

Moreover, studies consistently show that males are more likely to be lonely than females (Mahon et al., 2006; Ren et al., 2021) and did not recognize their loneliness (Cramer and Neyedley, 1998). Besides this, loneliness is proved to be a predictor of negative coping in females (Zhang et al., 2019). Therefore, sex differences might play a role in the association between loneliness and coping styles and, thus, affect depression.

**FIGURE 1:**
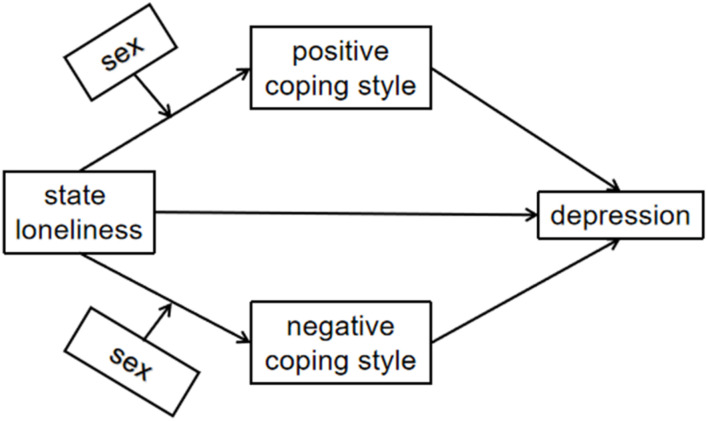
The hypothetical model.

The aim of our study was to explore the mediating effect of negative and positive coping styles on the relationship between state loneliness and depression as well as the moderating effect of sex on the association between loneliness and coping styles during COVID-19 quarantine. Based on previous findings, we hypothesized that loneliness could be predictive of depression during COVID-19 quarantine with coping style as a mediator. Moreover, we hypothesized that sex could be a moderator in the relationship between state loneliness and coping style during COVID-19 quarantine (Figure 1).

## Materials and Methods

### Participants

Participants in this study were recruited through social networks and were asked to fill out online questionnaires from February 14 to February 29, which was the peak of the COVID-19 pandemic and the strictest period of nationwide self-quarantine. A total of 337 questionnaires were collected. Participants from Guangdong Province accounted for 72.97%, and those from Hubei Province accounted for 10.51%. None of them were confirmed as COVID-19 cases. They were informed of the contents, purpose, and confidentiality principle. Informed consent was obtained, and then a unique code was given to participants. After eliminating 12 questionnaires that were missed, filled in incorrectly, or repeated, 325 valid questionnaires were finally obtained (effective rate: 96.44%). All participants were between the ages of 17–30 with a mean age of 20.65 (SD = 1.791), and 223 (68.62%) females were included. Participants were compensated with 15 RMB. The study was approved by the Ethics Committee of Shenzhen University.

### Measures

#### Patient Health Questionnaire-9

We modified the Patient Health Questionnaire (PHQ-9) to measure depression during the lockdown of the COVID-19 pandemic. The questionnaire had nine items to measure the frequency of depression. The questionnaire was scored from 0 = “almost never” to 3 = “nearly every day.” Examples of the items are “Do you have little interest or pleasure in doing things during the lockdown of the COVID-19 pandemic?” and “Are you feeling down, depressed, irritable, or hopeless during the lockdown of the COVID-19 pandemic?” If scored less than 4, it is treated as non-depressed; if scores ranged from 5 to 14, it is treated as mildly depressed; if scores ranged from 15 to 19, it is treated as moderately depressed, if scored above 20, it is treated as severely depressed. Cronbach’s coefficient of the scale was 0.89, indicating a good reliability and validity.

#### State Anxiety Inventory

To control for the state of anxiety, we modified the State Anxiety Inventory (SAI) to measure college students’ temporary state anxiety during the COVID-19 pandemic, which was compiled by Spielberger in 1980. Individuals were required to report their state of anxiety during the lockdown of the COVID-19 pandemic on a scale ranging from 1 = “none at all” to 4 = “very much.” Examples of the items are “Do you presently worry over possible misfortunes during the lockdown of the COVID-19 pandemic?” and “Do you feel self-confident during the lockdown of the COVID-19 pandemic?” Range of scores is from 20 to 80 with higher scores representing more severe states of anxiety.

#### State Trait Loneliness Scale

We used the State Trait Loneliness Scale (STLS) to assess the short-term loneliness of college students. This scale was compiled based on the loneliness scale (UCLA) and was more convenient than UCLA. The scale had 12 items with a five-point Likert scale ranging from 1 = “in fully agreement” to 4 = “totally disagree.” Examples of the items are “Are you short of companionship during the lockdown of the COVID-19 pandemic?” and “Do you feel your interests and ideas are different from those of people around you during the lockdown of that COVID-19 pandemic?” Higher scores indicate more intensity of state loneliness. Cronbach’s coefficient of the scale was above 0.88, indicating a good reliability and validity.

#### Simplified Coping Style Questionnaire

We used the Simplified Coping Style Questionnaire to measure coping style among college students. The questionnaire fully combines China’s cultural and population characteristics. It has 20 items with two dimensions: positive and negative coping styles. It is scored from 0 = “never use” to 3 = “frequently uses.” Items from 1 to 12 are used to assess the positive coping styles (e.g., “Getting rid of difficulties through work and study or some other activities during the lockdown of the COVID-19 pandemic” and “Looking for different ways to solve the problem during the lockdown of the COVID-19 pandemic”). Items from 13 to 20 were used to assess negative coping styles (e.g., “Trying to forget what gets you in trouble” and “Getting through your negative feelings and just letting them go”). A high score showed that this method was used frequently. Cronbach’s coefficient of the scale was 0.90.

### Statistical Analysis

Statistical analysis was conducted by IBM SPSS Statistics (version 26.0). An independent *t-*test was used to analyze sex differences on state loneliness, coping style, and depression. The mediating role of coping style and the moderating role of sex were analyzed by PROCESS macro. We also controlled for sex and anxiety as covariates. The bootstrapping method produces 95% bias corrected confidence intervals of these effects from 5,000 resamples of the data. Confidence intervals without zero indicate a significant effect.

## Results

### Depression, Loneliness, and Coping Style of College Students Under the COVID-19 Pandemic

Descriptive statistics are presented in Table 1. Of the participants, 58.46% suffered depression; 20.31% suffered moderate or more serious depression. In terms of sex, 31.38% of males reported they suffered from depression, and 68.62% of females did so. There was a significant effect of sex on depression (*t* = −2.18, *p* = 0.03, *d* = −0.26). Sex difference was not found on loneliness (*t* = −0.16, *p* = 0.88, *d* = −0.02), state anxiety (*t* = −0.77, *p* = 0.44, *d* = −0.09), positive coping style (*t* = −1.06, *p* = 0.29, *d* = −0.17), or negative coping style (*t* = −0.13, *p* = 0.9, *d* = −0.02).

**TABLE 1:** Descriptive statistics and correlations among key variables.

	Mean	SD	State loneliness	Positive coping	Negative coping	Depression
Age	20.65	1.791				
SAI	38.37	10.09				
State loneliness	28.44	6.80	1			
Positive coping	2.07	0.45	−0.35**	1		
Negative coping	1.77	0.48	−0.18**	–0.01	1	
Depression	6.11	4.71	0.44**	−0.35**	−0.19**	1

**p < 0.01. SAI, score of State Anxiety Inventory; SD, standard deviation.

To explore the regional differences on loneliness, we divided college students into different regional groups (students in Wuhan City, students outside Wuhan City in Hubei Province, students outside Hubei Province). Results showed that there was no significant regional difference on state loneliness during the lockdown (*F* = 2.48, *p* = 0.08).

### Correlation Between Loneliness, Depression, and Coping Style

Correlations for the measured variables, together with descriptive statistics are presented in Table 1.

There was a positive correlation between state loneliness and depression (*r* = 0.44, *p* < 0.01) and a negative correlation between state loneliness and positive coping style (*r* = −0.35, *p* < 0.01). Positive coping style was negatively predictive of depression (*r* = −0.35, *p* < 0.01). Meanwhile, negative coping style was negatively correlated with depression and loneliness (*r* = −0.18, *p* < 0.01, *r* = −0.19, *p* < 0.01). The correlation was not significant after controlling for anxiety.

Furthermore, we tested the predictive power of state loneliness on depression with linear regression. It was found that state loneliness could be an effective predictor of depression, and it accounted for 19% of the variation in depression (β = 0.44, *t* = 8.69, *p* < 0.001) with 95% CI: 0.233, 0.370.

### The Mediating Role of Coping Style Between State Loneliness and Depression

We found that sex did not moderate the relationship between loneliness and positive coping (95%CI: −0.01, 0.03), loneliness and negative coping (95%CI: −0.01, 0.02), positive coping and depression (95%CI: −2.04, 2.6), negative coping and depression (95%CI: −3.84, 0.16), or loneliness and depression (95%CI: −0.25, 0.16).

**FIGURE 2:**
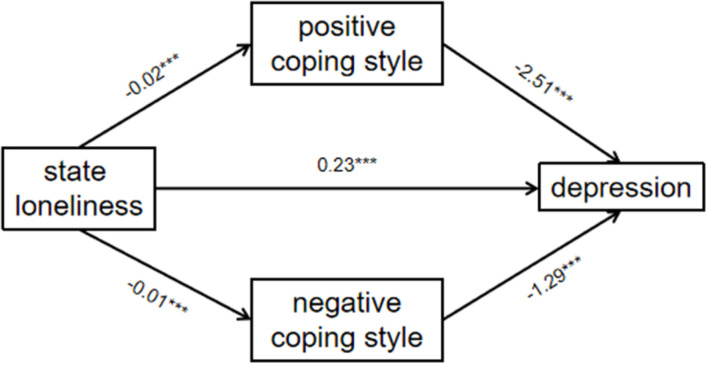
The pathway of coping style to state loneliness and depression ***p < 0.001.

The results showed that state loneliness could predict positive coping style (β = −0.02, *t* = −6.66, *p* < 0.001). State loneliness could predict depression (β = 0.23, *t* = 6.24, *p* < 0.001), and positive coping could predict depression (β = −2.51, *t* = −4.69, *p* < 0.001). It was indicated that positive coping was a significant mediator in the path of state loneliness affecting depression (Table 2, Figure 2).

**TABLE 2:** Regression analysis of state loneliness, positive coping style, and depression.

	Model 1 (Positive coping)			Mode 2 (Negative coping)			Model 3 (Depression)		
	β	SE	*t*	β	SE	*t*	β	SE	*t*
State loneliness	−0.02**	0.003	-6.66	−0.01**	0.004	−3.29	0.23**	0.04	6.24
Positive coping							–2.51	0.53	–4.69
Negative coping							−1.29**	0.48	–2.69
*R* ^2^	0.12			0.03			0.25		
*F*	44.41			10.8			36.3		

**p < 0.01.

State loneliness could predict negative coping style (β = -0.01, *t* = −0.003, *p* < 0.001). State loneliness could predict depression (β = 0.23, *t* = 6.24, *p* < 0.001). Negative coping could predict depression (β = −1.29, *t* = −2.69, *p* < 0.001). It was indicated that the mediating effect of negative coping was significant in the path of state loneliness affecting depression (Figure 2). After controlling for state anxiety scores and sex, our main results did not change.

A bootstrap method was used to explore effectiveness. The confidence interval of the results of the mediation test does not contain zero, further indicating the significance of the mediating of coping style. State loneliness affected the depression of youths with the total effects of 0.3 (95% CI: 0.23, 0.37), including the direct effect size of 0.23 (95% CI: 0.16, 0.3) and the indirect effect size through coping style of 0.07. At the same time, the indirectly mediating power of positive and negative coping were 0.06 (95% CI: 0.03, 0.1) and 0.01 (95% CI: 0.003, 0.035), respectively. Therefore, coping style mediated the relationship between state loneliness and depression during the lockdown, and the mediating effect accounted for 24.78% of the total effect.

## Discussion

This study was aimed to explore the relationships between loneliness, coping style, and depression and investigate the moderating role of sex between state loneliness and coping styles among youths under COVID-19 lockdown. We found that loneliness effectively predicted depression, and coping style played a mediating role, although sex did not moderate the relationship between state loneliness and coping styles. Sex only differed in depression, which was that females were more likely to be depressed during the lockdown. There were no sex differences in state loneliness and coping style. All results were not affected by the state of anxiety.

State loneliness during COVID-19 lockdown was positively predictive of depressive symptoms. This is consistent with previous studies (Lau and Kong, 1999; Cacioppo et al., 2010) as well as a recent meta-analysis (Erzen and Çikrikci, 2018). Lonely students could easily get into difficulties because of negative cognitive, and they were more likely to be depressed (Zawadzki et al., 2013). Adults experienced more loneliness during the quarantine, which can lead to poorer mental health (Creese et al., 2020). The previous study also revealed that depression was intensively correlated with loneliness in the elderly, which was produced when an individual was unable to form a good attachment to others (Peerenboom et al., 2015). Besides this, patients with cancer who experience more loneliness also have an increased risk of depression during COVID-19 (Gallagher et al., 2020). Adding to these, our results indicate that youths who are in lockdown at home may experience depressive symptoms as well as loneliness.

Coping style played a partial mediating role between relationships and loneliness. On one hand, loneliness could negatively predict positive coping style, and positive coping style could negatively predict depression. Individuals with low loneliness tend to use positive coping styles and avoid negative coping styles (Quan et al., 2014), and high loneliness would reduce proactive behavior and the desire to explore the environment (Bolger and Amarel, 2007). Moreover, social supports from family and friends were negative predictors of low loneliness (Salimi and Bozorgpour, 2012). Hence, our results suggest that college students with low loneliness may relate to family support and understanding of the lockdown policy under COVID-19, and they would be more likely to adopt positive coping styles to deal with negative emotions, such as depressive symptoms. On the other hand, negative coping styles also mediated the relationship between state loneliness and depressive symptoms during the lockdown. A previous study reports that loneliness was related to an increased use of passive coping strategies, which involve behaviors such as not dealing with problems and relate to maladaptive psychological outcomes (Vanhalst et al., 2012). Negative response styles, such as rumination, are also associated with depressive symptoms and mediate the effect of loneliness on depression (Zhang et al., 2019). Contrary to these results and our expectations, negative coping styles were negatively correlated with loneliness and depressive symptoms during the COVID-19 lockdown. This inconsistency may result from the negative coping strategies involved in our study being different from the previous negative response style or passive coping. For instance, strategies such as distracting from the problem or persuading oneself to accept the situation contain the component of cognitive reconstruction for the sake of actively solving the problem, which are different from passive coping or negative rumination responses in previous studies. Studies already show that distraction and cognitive reconstruction were effective in relieving negative emotions, including depression (Goldin et al., 2008; Cheng et al., 2009; Dörfel et al., 2014). Therefore, although these negative coping strategies may not directly resolve the stressors, it may distract youths from the pandemic and have some effect on alleviating the negative emotion under the COVID-19 lockdown. Together, our results imply that not positive coping would help youths to adapt to healthy mental states and effectively deal with the pandemic threats, but negative coping would help to relatively alleviate maladaptive emotions during the lockdown. It would be of great importance to improve college students’ ability to actively deal with the stressful situation.

Sex did not moderate the relationship between loneliness and coping style during the lockdown. Previous research reports that compared with males, female adolescents with high loneliness were more likely to have a negative response style in general, which was calculated by the ratio of rumination to problem solving and distraction (Zhang et al., 2019). However, in our study, to identify the different role of positive and negative coping, we included more negative coping strategies than rumination and investigated the moderate effect of sex on loneliness to negative and positive coping, respectively. Nonetheless, we did check the sex effect on the relationship between loneliness and overall coping style (i.e., ratio of negative to positive coping) and found no moderate effect of it. Together with the findings that sex differences are found in depressive symptoms but not loneliness or use of coping styles, we speculate that the homogeneous nationwide lockdown policy during the pandemic may result in non-discriminatory social contacts for both males and females, thus leading to the same level of loneliness in them, and promote the coordinative ability to use coping strategies to deal with stressful situation.

## Implications

It is of great importance to improve people’s mental health with COVID-19 pneumonia spreading globally, especially for college students with unbalanced physiological and psychological characteristics. Our research shows that coping style mediates the relationship between loneliness and depression, which suggests that a suitable coping style or strategy plays an important role in preventing and intervening in the depressed state among college students. First, given the lockdown situation during the pandemic and the evidence that more social connection predicts less depression (Jose and Lim, 2014), it would be helpful for college students to interact with friends through social media and communicate with the family frequently to meet their social needs and to reduce their solitude. Second, it would be beneficial if they could adjust the goal of interpersonal interaction through cognitive reappraisal. For instance, it may be inevitable to be socially restricted during the lockdown. Therefore, it would be good for college students to understand and support the lockdown policy and to hold a bright view of the pandemic, which may help to relieve negative emotions, such as loneliness and the state of depression.

## Limitations

There were some limitations in our study. As we adopted a self-report approach in our cross-sectional study, it might be difficult to indicate the causality of the variables. Hence, it would be interesting to investigate the causality through longitudinal design in the future. Moreover, although regional differences on state loneliness during lockdown were not significant in our study, a future study may include more objective measurements on mental states during the COVID-19 pandemic, such as lockdown levels and pandemic threat, as indicated in a recent study (Zheng et al., 2020).

## Conclusion

Our research explored the relationship among depression, state loneliness, and coping style and sex differences during the COVID-19 pandemic. State loneliness was a significant predictor of depression, and both positive and negative coping styles could partially mediate the relationship between state loneliness and depression under the lockdown. Our findings shed light on public mental health intervention to youths.

## Data Availability Statement

The raw data supporting the conclusion of this article will be made available by the authors, without undue reservation.

## Ethics Statement

The studies involving human participants were reviewed and approved by Ethics Committee of Center for Brain Disorders and Cognitive Science. The patients/participants provided their written informed consent to participate in this study.

## Author Contributions

YZ and LH did the survey and data analyses. HA and YL designed the study and wrote the manuscript. All authors contributed to the article and approved the submitted version.

## Conflict of Interest

The authors declare that the research was conducted in the absence of any commercial or financial relationships that could be construed as a potential conflict of interest.

## Publisher’s Note

All claims expressed in this article are solely those of the authors and do not necessarily represent those of their affiliated organizations, or those of the publisher, the editors and the reviewers. Any product that may be evaluated in this article, or claim that may be made by its manufacturer, is not guaranteed or endorsed by the publisher.
